# Patient Perspectives and Outcomes in Adult Hypospadias Repair

**DOI:** 10.7759/cureus.44021

**Published:** 2023-08-24

**Authors:** Mukesh Chandra Arya, Rambir Singh, Pranjal Moharjal, Prashant Gupta, Ankur Singhal, Aayush Mittal, Pradyot Shahi, Somit Kumar, Ajay Gandhi, Yogendra Shyoran

**Affiliations:** 1 Urology, Sarder Patel Medical College, Bikaner, IND; 2 Urology, Sawai Man Singh (SMS) Medical College, Jaipur, IND

**Keywords:** hypospadias repair, chordee, urethrocutaneous fistula, penile torsion, meatal stenosis, adult hypospadias

## Abstract

Background

Hypospadias is a common congenital anomaly that needs repair at an early age (six months to one year). Ironically, many cases in India present late due to a lack of healthcare facilities, poverty, and illiteracy. Adult patients are different from children as they are aware of their genitalia. They are concerned with the aesthetics and, predominantly, the potency. In this study, we present the perspectives and outcomes of 111 adult cases of hypospadias.

Methodology

In this retrospective study conducted between January 2010 and December 2020, 111 patients aged more than 14 years who were diagnosed with hypospadias of any level with or without mild-to-moderate chordee were included. Hypospadias repair using a tubularized incised plate (TIP) urethroplasty technique was performed, and patients after surgery were followed up at three months, six months, and 12 months for any complications with physical examination, uroflowmetry, and patient-related outcomes (PROs).

Results

Age varied from 14 years to 32 years (mean = 19.88 years, SD = 5.93). The most frequent meatus positions after chordee adjustment were distal (n = 64, 57.65%), middle (n = 25, 22.52%), and proximal (n = 22, 19.82%). Among these, four patients had penoscrotal transposition. Chordee was present in 65.7% (n = 73) of the cases. (<30° in 38.7%, n = 43; 30°-60° in 23.4%, n = 26, and >60° in 3.6%, n = 4). Chordee was corrected using many techniques, including ventral corporotomies. Urethroplasty was done using TIP and spongioplasty in 89% (n = 99), and one patient underwent inner preputial onlay flap urethroplasty. Snodgraft was used to augment the urethral plate in 10 cases. The success rate of one-stage surgery was 74.77% in our series, which significantly correlated with PROs. Uroflow varied from 12 mL/second to 18 mL/second, and in the majority of the cases, the flow rate improved over time. The most common complication was urethrocutaneous fistula in 11 (11.8%) patients, followed by glanular dehiscence in nine (8.1%) patients.

Conclusions

Adult patients undergoing primary hypospadias repair generally show good outcomes. Patients can have an acceptable mild degree of residual chordee and torsion, which correlate well with PROs. In our series, hypospadias fistula was the most common complication of hypospadias surgery, followed by glanular dehiscence.

## Introduction

Hypospadias is a common male genitourinary anomaly (with a prevalence of one in 200 live births) requiring surgery at six months to one year of age [1]. Contrary to the Western world, in developing countries, due to poverty and ignorance, such patients (30% in our series) present in adulthood. In contrast to children, adults are worried about aesthetics and potency. The outcomes of hypospadias repair are improving with the advancement of surgical techniques and meticulous repair [2]. Although multiple techniques and their modifications have been described for hypospadias repair, no single technique is applicable to all cases. Therefore, surgeons should be well-versed with various techniques according to the needs of the case. However, failed cases continue to suffer from physical and psychological disabilities [3]. Our article primarily discusses the management of adults with hypospadias, who were affected both functionally and psychosexually. Ideally, these individuals need long-term follow-up until they can sexually cohabitate with satisfaction [4].

## Materials and methods

Study design and participants

This is a hospital record-based, retrospective analytical study. All hypospadias cases 14 years of age or older (n = 111) who visited our department from January 2010 to December 2020 were included in this study.

Inclusion and exclusion criteria

Primary hypospadias cases were included in this study. On the other hand, hypospadias cases below 14 years of age (n = 198), secondary cases with prior hypospadias (n = 23), those with isolated penile torsion (n = 12), and those lost to follow-up (n = 33 patients) were excluded.

Sample collection

**Figure 1:**
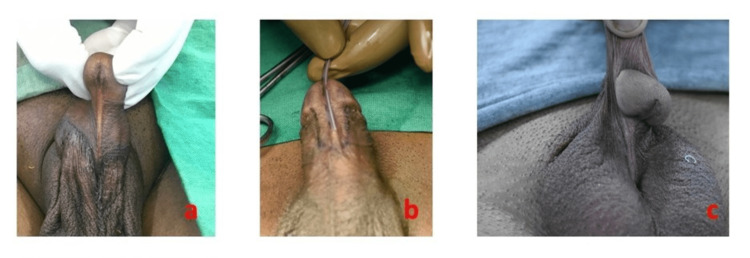
Different types of adult hypospadias. (a) Adult hypospadias with significant chordee. (b) Mid-penile adult hypospadias. (c) Another mid-penile adult hypospadias.

In this study, age at surgery, the position of the meatus, the associated chordee, the quality of the urethral plate (Figure 1), surgical techniques, and outcomes, including complications and success rate, were studied.

Surgical procedure

Anesthesia and Instruments

All patients were operated on in a supine position under general anesthesia, in addition to caudal anesthesia in proximal hypospadias cases. Microsurgical instruments, ophthalmic blades, bipolar cautery, and magnification loupes were utilized during the surgery.

Cannulation and Stay Sutures

The urethral stent size varied from 8 Fr to 10 Fr, depending on the age of the patient. The length of the infant feeding tube to be inserted was measured from 3 cm above the symphysis pubis to the external meatus to prevent coiling and knotting and reduce detrusor spasms. During difficult cannulation, it was coaxially put over a 0.035-inch Terumo guidewire. In case of failure, a guidewire was placed using a 6/7.5-Fr ureterorenoscope (URS). Stay sutures (5-0 prolene) were placed before embarking on the suture in the sagittal plane from the meatus to ease dissection. Prolene sutures do not leave a stitch mark.

Incision and Degloving

A biopsy was obtained for patients with meatal stenosis to rule out balanitis xerotica obliterans (BXO) [5]. The urethral plate was measured with a ruler at the level of the corona. The circumcoronal incision was extended up to the midglans. The hypoplastic urethra (intimately blended with skin) was incised in the midline. The incision was vertically deepened until Buck’s fascia so that the underlying dartos remained attached to the skin. This allowed us to close the ventral dartos as a separate buffering layer and reduced skin ischemia. Degloving was done up to the penoscrotal and penopubic areas.

Chordee Correction

After degloving, the bifurcated corpus spongiosum was mobilized. Gitte’s test was done to assess the chordee [6]. A mild chordee was corrected by a 5-0 prolene suture. Dorsal penile plication was done at the site of maximum chordee. In the case of chordee >30°, from 3 to 9 o’clock, the outer layer of tunica albuginea was incised. Using an ophthalmic blade three ventral corpotomies were made 5 mm apart. In cases of severe chordee, ventral penile skin was short; therefore, dorsal penile skin was brought ventrally by Byar’s flap.

Tubularized Incised Plate/Snodgraft

The urethral plate was tubularized using Vicryl 4-0/5-0 longitudinal subcutaneous sutures. If the urethra was narrow, it was augmented by buccal mucosa or inner prepuce inlay. Glansplasty was done in all cases using 5-0 subcuticular sutures.

Spongioplasty

A bifurcated corpus spongiosum distal to the meatus was mobilized off the corporal bodies and closed in the midline (spongioplasty).

Buffering Layers

In all cases, ventral dartos were closed in addition to dorsal dartos (n = 22) and tunica vaginalis blanket wrap (n = 23).

Skin Closure and Correction of Torsion

Correction of torsion (n = 7) was done by rotating the skin at the corona, and the skin was closed with 5-0 Vicryl after placing a glove drain.

**Figure 2:**
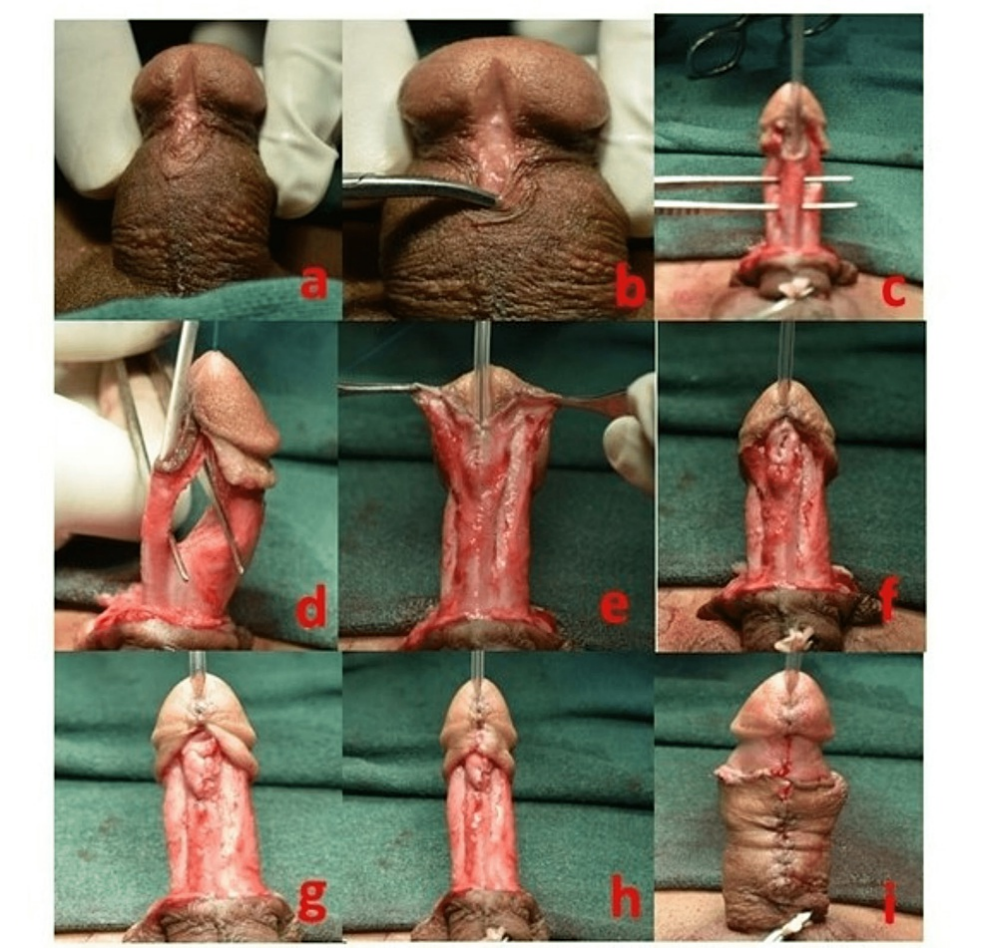
Steps of repair for adult hypospadias. (a) Adult distal penile meatus. (b) Identification of the meatus and hypoplastic urethra. (c) Urethral mobilization in the longitudinal view. (d) Urethral mobilization in the coronal view. (e) Glans wings and bifurcated corpus spongiosum. (f) Urethral closure and spongioplasty: first layer. (g) Spongioplasty: second layer. (h) Glansplasty. (i) Closure of the skin.

**Figure 3:**
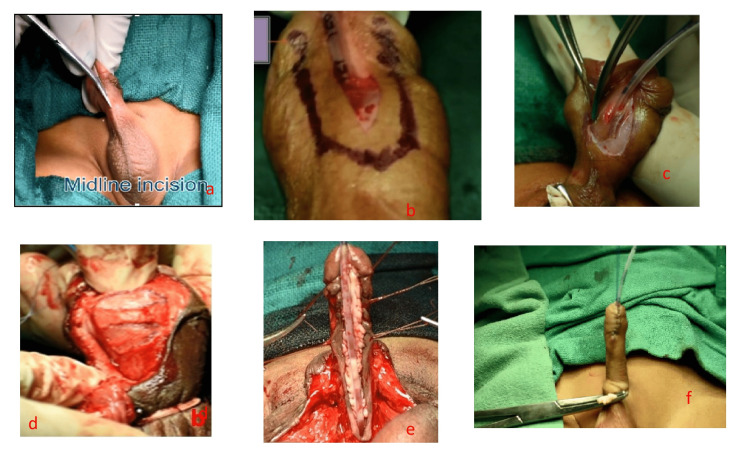
Operative steps. (a) Midline incision of the hypoplastic urethra. (b) Incision up to the mid-glans. (c) Vertical deepening of the incision up to Buck’s fascia. (d) Ventral corporotomies. (e) Augmented urethral plate with two BMGs. (f) Prepucioplasty.

Figure 2 shows the steps for adult hypospadias repair, and Figure 3 shows the operative steps.

Finally, prepucioplasty was done in six cases. The mean operative time was 91.68 minutes, with an SD of 11.16 (60-120 minutes).

Patients received third-generation cephalosporins and aminoglycosides for three to five days. The drain tube was removed on day three. The mean hospital stay was 7.08 days, with an SD of 1.12 days (5-10 days). The urethral stent was kept for two weeks.

Data collection and follow-up

After permission from the institutional ethical committee, we collected and analyzed data from departmental digital records.

Patient-related outcomes

**Figure 4:**
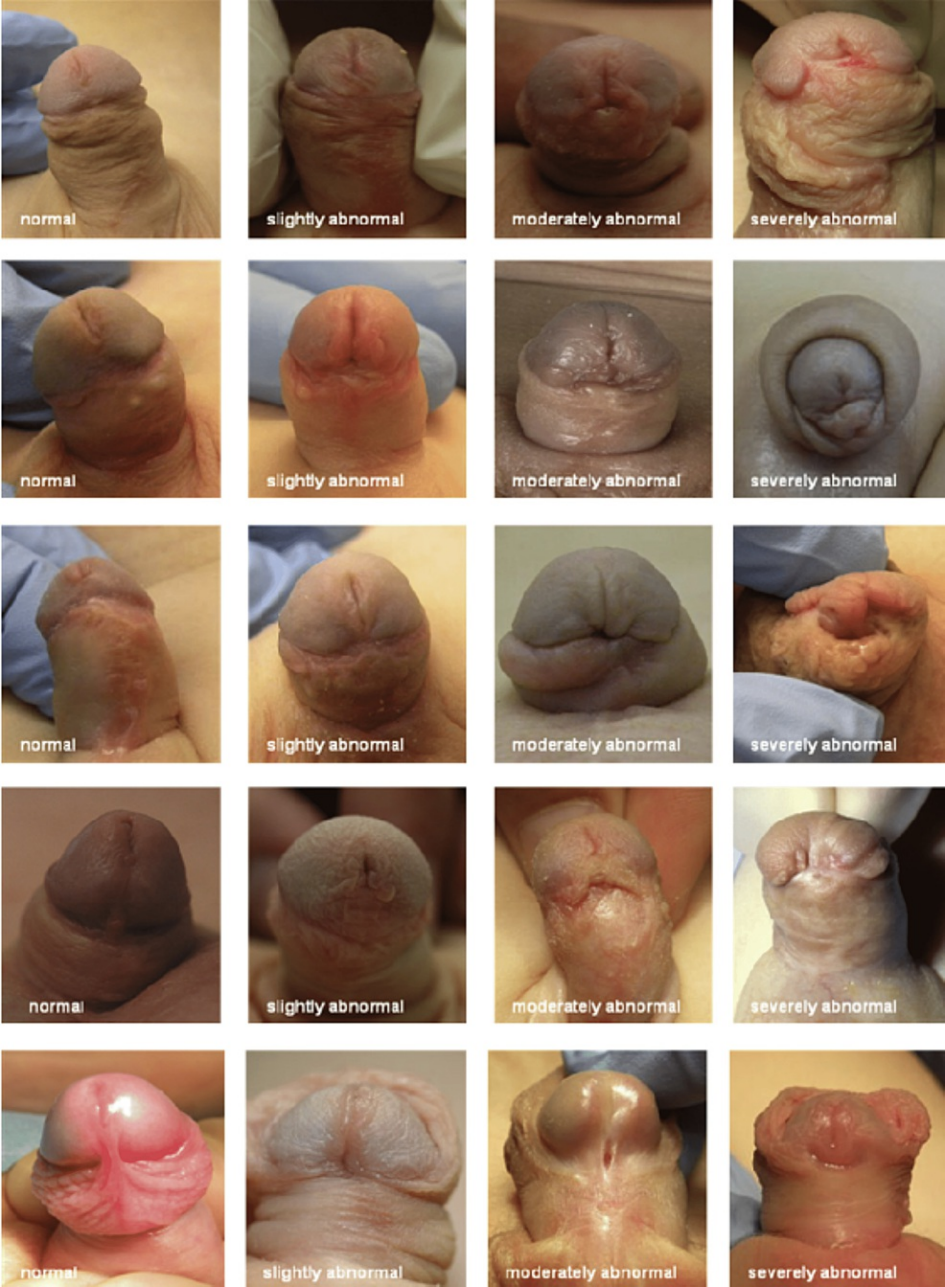
Standard images from the Hypospadias Objective Penile Evaluation (HOPE) score.

Patient-related outcomes (PROs) were calculated using the Hypospadias Objective Penile Evaluation (HOPE) criteria (Figure 4) and studied [7]. The following five variables were included: position of the meatus, the shape of the meatus, the shape of the glans, the shape of the skin, residual torsion, and chordee. These variables were assessed and grouped into six questions, each with a reference score. The maximum score was 10, and the minimum score was one. The combined score ranged from 6 to 60. A cutoff of 6-23 was arbitrarily taken for severe abnormalities; 24-41 for moderately abnormal which were considered negative outcomes; and scores between 42-54 for mildly abnormal and more than 54 were classified as good or satisfactory results, as reported by patients. All patients were followed up for at least 12 months.

Statistical analysis

We used the chi-square test for qualitative data and the unpaired Student’s t-test for quantitative data to deduce p-values.

## Results

Among the 111 patients, 58 and 53 patients were aged up to 20 or older, respectively. The mean age of the patients in our study was 19.88 years (SD = 5.9) (Table 1). The most common meatal position in adult cases was distal (n = 64, 57.65%), followed by middle (n = 25, 22.52%) and proximal (n = 22, 19.82%). Among these, four patients had penoscrotal transposition. The surgical techniques employed in this study are presented in Table 2.

**Table 1:** General characteristics of the patients.

	N (%)
Age (in years)	
14–20	58 (52.3%)
20–30	44 (39.6%)
>30	9 (8.1%)
Meatal position	
Distal	64 (57.65%)
Middle	25 (22.52%)
Proximal	22 (19.82%)
Chordee in degree	
No chordee	38 (34.2%)
<30°	43 (38.7%)
30°–60°	26 (23.4%)
>60°	4 (3.6%)
Urethral plate	
Healthy	62 (55.9%)
Adequate	37 (33.33%)
Narrow	8 (7.20%)
Indistinct	4 (3.6%)

**Table 2:** The various methods of repair utilized in the study.

		Frequency	Percentage
Types of procedure	Tubularized incised plate (Snodgrass) and spongioplasty	99	89
	Snodgraft and spongioplasty	10	9
	Preputial island onlay flap	1	0.9
	Two-stage	1	0.9
Total		111	100
Penoscrotal transposition	Heineke-Mikulicz	2	3.6
	Glenn Anderson	2	
Buffering layer			
	Dorsal dartos	22	19.8
	Tunica vaginalis flap	23	20.7
Urethral plate			
	Adequate	99	89
	Inadequate	12	11
Prepucioplasty			
	No prepucioplasty	105	94.6
	Prepucioplasty done	6	5.4

Chordee was assessed after the Gittes test, and 73 patients had varying degrees of chordee, of whom 43 patients had mild chordee (<30°), 26 patients had moderate chordee (30°-60°), and four patients had severe chordee (>60°). Various techniques were used for the correction of chordee, including penile degloving (PD), urethral mobilization (UM), ventral corporotomies (VC), and dorsal plication (DP), as described in Table 3. A mild degree (<30°) of penile torsion was present in a small number of patients (n = 7), which was corrected by rotating skin at the corona with satisfactory results.

**Table 3:** Chordee correction techniques.

Chordee correction technique	Number of patients	Percentage
Penile degloving	11	9.9
Penile degloving and urethral mobilization	29	26.1
Penile degloving, urethral mobilization, and ventral corporotomy	6	5.4
Penile degloving, urethral mobilization, ventral corporotomy, and dorsal plication	25	22.5
Penile degloving, urethral mobilization, dorsal plication	2	1.8
Total	73	100.0

Various approaches for urethral tube reconstruction included tubularized incised plate (TIP), TIP and spongioplasty, and the preputial island onlay flap [8]. Penoscrotal transposition in four patients was corrected by horizontal incision and longitudinal closure in two patients and using the Glenn-Anderson technique in the remaining two patients [9]. Urethroplasty was staged in one patient (Table 2). Second-layer closure was done in all cases using ventral dartos with additional layers of dorsal dartos in 22 cases and the tunica vaginalis flap (TVF) in 23 cases.

Many patients required urethral plate augmentation (n = 10) by Snodgraft using either buccal mucosa (n = 8) or inner prepuce (n = 2). Prepucioplasty was performed in six patients. In none of the cases, the biopsy turned out to be BXO.

The failure was evaluated based on the type of hypospadias, degree of chordee, quality of the urethral plate, and buffering layer, along with their p-value. PROs were evaluated using the HOPE criteria, and their concordance with the success rate is shown in Table 4. The postoperative complications in the form of glans dehiscence, meatal stenosis, UCF, stricture, etc. are shown in Table 5, along with the concordance of success rates with PROs.

**Table 4:** The outcomes of success and failure. TVF = tunica vaginalis flap; HOPE = Hypospadias Objective Penile Evaluation

	Success			Success percentage	P-value
	Meatal position	Success (%)	Unsuccessful (%)		
Type of hypospadias					
	Distal	54 (84.3)	10 (15.6)	84.37	>0.05
	Middle	18 (72)	7 (28)	72	
	Proximal	13 (59.1)	9 (40.9)	59.1	
Success rate		85 (76.57)	26 (23.43)	76.5766	
Chordee					
	No chordee	31 (81.57)	7 (18.43)	81.57895	>0.05
	<30°	34 (79.06)	9 (20.94)	79.06977	
	30°–60°	18 (69.23)	8 (30.77)	69.23077	
	>60°	2 (50)	2 (50)	50	
Urethral plate					
	Healthy	48 (77.41)	14 (22.59)	77.41935	>0.05
	Adequate	32 (86.48)	5 (13.52)	86.4864	
	Narrow	4 (50)	4 (50)	50.0000	
	Indistinct	1 (25)	3 (75)	25.0000	
Buffering layer					
	dorsal dartos	15 (68.18)	7 (31.72)	68.18182	>0.05
	TVF	20 (86.95)	3 (13.05)	86.95652	
Patient-related outcomes HOPE score				Concordance	
	>54	13 (100)	0 (0)	100	>0.05
	42–54	70 (97.22)	2 (2.78)	97.22	
	24–41	2 (8.65))	21 (91.35)	8.65	
	6–23	0 (0)	3 (100)	0	

**Table 5:** Complications in our study.

Complications	Present	Percentage (%)
Glans dehiscence	9	8.1
Meatal stenosis	5	4.5
Fistula	11	9.9
Stricture	5	4.5
Residual chordee	0°–10°: 15	13.5
	10°–30°: 0	0
Torsion	0°–10°: 5	4.5
	>10°: 0	0
Retrusive meatus	1	0.9
Preputial dehiscence	1	0.9

## Discussion

The mean age of the patients in our study was 19.88 years, which is comparatively lower than that reported by Hoy et al. [10]. The reasons for this late presentation were poverty, lack of awareness, difficult access to healthcare, and low literacy rates compared to other parts of the world. A significant number of patients in our study visited us before getting married. Many techniques can be used for the reconstruction of a normal phallus and depend on the surgeon’s preferences and comfort with that technique. Similarly, the choice of graft or flap for urethral augmentation and variations in chordee correction techniques also varied [11].

Successful reconstruction of the phallus not only involves the surgeon’s aspects of the repair, i.e., normal voiding, fornication, and cosmesis, but also the views of the patients and their assessment of the phallus, especially in adulthood [12]. Patients with successful surgical outcomes may still be unhappy due to their fear of sexual performance and their partner’s perspective. Such patients may report their phallus to be abnormal and remain dissatisfied. Spongioplasty helped reduce fistulas and diverticula apart from strengthening the neourethra. Chordee correction techniques included PD in 11 patients; PD and UM in 29 patients; PD, UM, and VC in six patients; PD, UM, VC, and DP in 25 patients; and PD, UM, and DP in two cases. Urethroplasty techniques included TIP with and without spongioplasty in 99 patients, a staged procedure in one patient due to an indistinct urethral plate, Snodgraft in 10 patients, and an island flap in one patient. Four patients underwent repair of penoscrotal transposition via Heineke-Mikulicz and Glenn-Anderson techniques in two patients each. Ganuloplasty was done in all cases using Vicryl 5-0 subcuticular sutures. The adequate glans wing ensured that glanuloplasty sutures did not constrict the neourethra.

The urethral plate was healthy and adequate in 62 and 37 patients, respectively, and urethroplasty was done using TIP. The urethral plate was narrow and indistinct in eight and four patients, respectively, and was augmented using Snodgraft in 10 cases. Similar to our series, Shimotakahara et al. [13] reported a random comparison between the Snodgraft and Snodgrass procedures, reporting a lower success rate for the Snodgraft than that following the TIP (p < 0.05 and p < 0.01, respectively). At each follow-up visit, uroflowmetry was done. Qmax varied in individual patients at various visits. However, there was an overall improvement in Qmax over the follow-up period (Table 6).

**Table 6:** Uroflowmetry (Qmax) at follow-up (n = 85).

Visits	12–14 mL/second	14–18 mL/second
3 months	66 (77%)	19 (33%)
6 months	49 (57%)	36 (43%)
12 months	40 (47%)	45 (53%)

**Figure 5:**
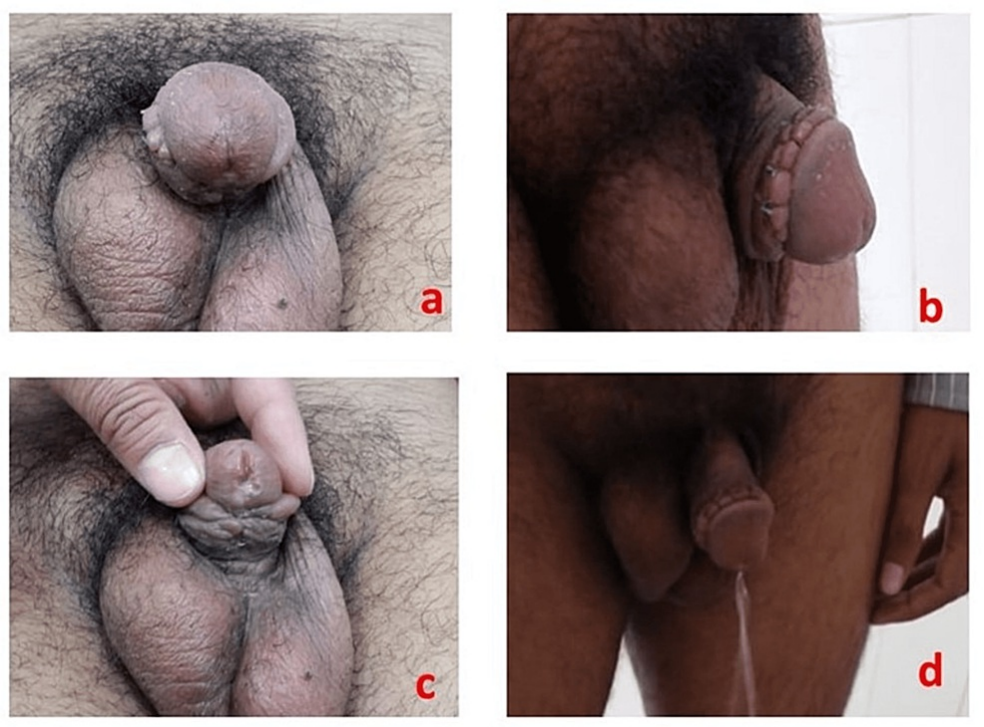
Postoperative follow-up. (a) Front view. (b) Lateral view. (c) Ventral surface view. (d) Voiding film.

The success rate was defined as patients not needing any further intervention and having normal functional aspects (Table 4, Figure 5). In our study, the overall success rate was 74.77% (n = 83). In this group, mild residual chordee of <10° and mild torsion of <10° were present in 15 and 5 patients, respectively, but did not require any intervention. On the other hand, 26 patients had failed repairs that required intervention, and two patients were satisfied with their outcomes. It was slightly higher compared to a study by Al Taweel et al. [14] in 2017, which was 71%. The most common complication was urethrocutaneous fistula (UCF) in 11 (11.8%) patients, followed by glanular dehiscence in nine (8.1%) patients. Other complications included meatal stenosis and stricture in five patients each. Many of these had a combination of complications, e.g., fistula and stricture. Moreover, one out of six patients who underwent a prepucioplasty had preputial dehiscence. A retrusive meatus was also present in one patient. In our study, the fistula rate was low compared to the study by Al Taweel et al [14]. This may be explained by the use of additional barrier layers in the form of the tunica vaginalis, dorsal dartos, and ventral dartos. The most common complication was meatal stenosis in a study by Pfistermuller et al. [15] and Jayanthi et al. [16] which was in contrast to our study. Patients who had glanular dehiscence underwent glanuloplasty. Patients with UCF underwent fistula closure with an additional barrier layer in the form of TVF. Meatal stenosis is the worst complication, as it puts the proximal repair at risk and should be avoided by careful meatoplasty. Meatal calibration after removal of the urethral stent plays an important role in the follow-up to detect it. One patient who had preputial dehiscence needed circumcision. A successful second-stage urethroplasty was done in one patient who was staged due to an indistinct urethral plate after six months. Two patients with penoscrotal transposition underwent Glenn-Anderson repair six months later.

The success rate varied with the position of the meatus, and patients with distal meatus had the best results (84.37%), followed by middle hypospadias (72%). The worst outcomes were reported for proximal cases, with the success rate dropping to 59.1%. However, there was no statistical significance as the comparison groups were not even. There were fewer proximal hypospadias cases to produce any statistical significance. Similarly, the success rate varied with the severity of chordee, with the best outcomes seen in patients with mild or no chordee (79% and 81%, respectively), and bad success rates were noted with moderate or severe chordee (69% and 50%, respectively), but without any statistical significance. Further, urethral plate quality and the use of buffering layers did not lead to statistically significant (p > 0.05) differences in surgical outcomes. In contrast, in a study by Chung et al. [17], fistula formation was related to the location of the meatus with statistical significance.

Adult hypospadias is an important subject in the field of hypospadiology, as adult patients have an awareness of their genitalia and have sexual concerns in addition to structural and functional abnormalities in their phallus [4]. Management of adult cases should be done keeping in mind their psychological state with appropriate preoperative counseling of their urinary stream and sexual performance, in addition to aesthetic appearance. This study deals primarily with the evaluation of PROs and their concordance with surgical outcomes.

According to the HOPE score, 13 patients had a score of >54, and 70 had a score of 42-54 and were considered successful (74.77%). The remaining 28 patients had unsuccessful PROs. However, among these two patients with moderate abnormalities, scores of 24-41 were satisfactory. Overall, surgical outcomes correlated well with PROs.

The literature on adult hypospadiology is scarce. Al Taweel et al. [14] reported a 71% success rate in their series of primary hypospadias after the first surgery, while in a study by Adayener et al. [18], the success rate was 91.3% for primary adult cases and was comparatively higher to a similar series of hypospadias repair by Ullah et al. [19], who reported successful outcomes in 52 (69.33%) patients. Hemant et al. reported a success rate of 63.16% in primary adult hypospadias cases [20]. Rashed et al. reported a success rate of 74% in primary cases. They also compared results with children and found a success rate of 95.5% in children [21].

This study had some limitations. This is a single-institution retrospective study. Due to the short follow-up (12 months), an evaluation regarding potency was not possible. Studies with a larger number of patients are needed to draw further conclusions.

## Conclusions

The majority of hypospadias cases can be repaired in one stage with satisfactory outcomes. The use of the TIP with or without spongioplasty and additional buffering layers in cases with long urethral tubes helped decrease the fistula rates. The use of grafts nowadays has decreased complication rates when compared with flaps. A straight phallus with the ability to perform intercourse satisfactorily and without causing any risk to the upper tract and a good urinary stream may require multiple surgeries due to postoperative complications as well as the necessity of staged repair. PROs should be the standard of care and may differ from surgeons’ perspectives as there could be significant discordance between them.

The results of hypospadias surgery in our series are comparable to others; nevertheless, it is recommended to follow these patients until they engage in sexual activity successfully including the partner’s perspective. With the wider availability of medical care and improvements in literacy and economic status in India, hopefully, more patients will present at the recommended age of repair in the future.
